# Creative foraging: An experimental paradigm for studying exploration and discovery

**DOI:** 10.1371/journal.pone.0182133

**Published:** 2017-08-02

**Authors:** Yuval Hart, Avraham E. Mayo, Ruth Mayo, Liron Rozenkrantz, Avichai Tendler, Uri Alon, Lior Noy

**Affiliations:** 1 The Theatre Lab, Weizmann Institute of Science, Rehovot, Israel; 2 Department of Molecular Cell Biology, Weizmann Institute of Science, Rehovot, Israel; 3 Paulson School of Engineering and Applied Sciences, Harvard University, Cambridge, MA, United States of America; 4 Department of Psychology, The Hebrew University of Jerusalem, Jerusalem, Israel; 5 Department of Neurobiology, Weizmann Institute of Science, Rehovot, Israel; Fordham University, UNITED STATES

## Abstract

Creative exploration is central to science, art and cognitive development. However, research on creative exploration is limited by a lack of high-resolution automated paradigms. To address this, we present such an automated paradigm, the creative foraging game, in which people search for novel and valuable solutions in a large and well-defined space made of all possible shapes made of ten connected squares. Players discovered shape categories such as digits, letters, and airplanes as well as more abstract categories. They exploited each category, then dropped it to explore once again, and so on. Aligned with a prediction of optimal foraging theory (OFT), during exploration phases, people moved along meandering paths that are about three times longer than the shortest paths between shapes; when exploiting a category of related shapes, they moved along the shortest paths. The moment of discovery of a new category was usually done at a non-prototypical and ambiguous shape, which can serve as an experimental proxy for creative leaps. People showed individual differences in their search patterns, along a continuum between two strategies: a mercurial quick-to-discover/quick-to-drop strategy and a thorough slow-to-discover/slow-to-drop strategy. Contrary to optimal foraging theory, players leave exploitation to explore again far before categories are depleted. This paradigm opens the way for automated high-resolution study of creative exploration.

## Introduction

Creative exploration and discovery processes are defined as the search for novel and valuable elements within a set of constraints. Creative exploration is central for many fields, including art [1], design [2] and science [3]. More generally, it is an integral part of cognitive development [4,5]. Despite progress achieved in understanding creative search processes from different perspectives [6–14], much is still unknown about their underlying dynamics and mechanisms.

One obstacle for understanding creative exploration is the lack of automated and high-resolution experimental paradigms. For example, one of the most commonly used tests for creativity is the alternate usage task (AUT) [15]. In the AUT participants are asked to come up with creative uses for common objects (e.g., a ping-pong ball). Participants show exploration patterns such as switching between categories of solutions [12]. This test is coded manually, a laborious process which limits the extent of feasible studies. The test is difficult to analyze and model because the space of possible solutions is undefined (how many different usages for a ping-pong ball exist?) and has no natural notion of similarity between different solutions. Other common creativity tests share these limitations [16].

More fundamentally, AUT and other common creativity tests record only the discovered solutions and not the intermediate steps leading from one solution to another. Thus, they do not allow insight into the process of exploration. Several studies attempted to map the processes leading to creative solutions by recording verbal transcripts of thought processes—the ‘think aloud’ method [2,17]. However, these methods are difficult to code and quantitatively analyze.

Here, we introduce a new paradigm for creative exploration that allows automated tracking of the search process in a space of possibilities that is large yet well-defined. To achieve this, we borrow techniques from the study of human foraging.

In a series of studies Hills, Todd and colleagues used a spatial foraging task and a letter puzzle task to experimentally study human foraging behavior [18–21]. These experimental foraging tasks employed solution spaces which are finite and well-defined [22]. For example, in the spatial foraging task participants searched for resources on a grid of locations. This task records intermediate steps between solutions and has a clear distance metric, features that allow the foraging behavior to be quantitatively analyzed and mathematically modeled using optimal foraging theory (OFT) [23].

In order to develop a similar task for creative search, we need to address one key difference between foraging and creative search. In the foraging tasks, the goal is to find as many solutions as possible; in creativity tests the goal is two-fold: (i) to find many solutions (fluency) (ii) of high quality (novel and valuable). In other words, whereas the value of the different solutions in the foraging tasks is constant (for example, all foraged pixels in the spatial foraging task are equally valuable), the distribution of solution values in creativity tests is skewed, with many low-quality solutions (boring or non-feasible) and only a handful of high-quality creative solutions.

We present a paradigm for creative foraging in which players seek solutions that they find interesting and beautiful, embedded within a well-defined search space. The *creative foraging game* enables high-resolution measurement of creative, intrinsically motivated search in the framework of visual exploration and discovery [24–28].

**Fig 1:**
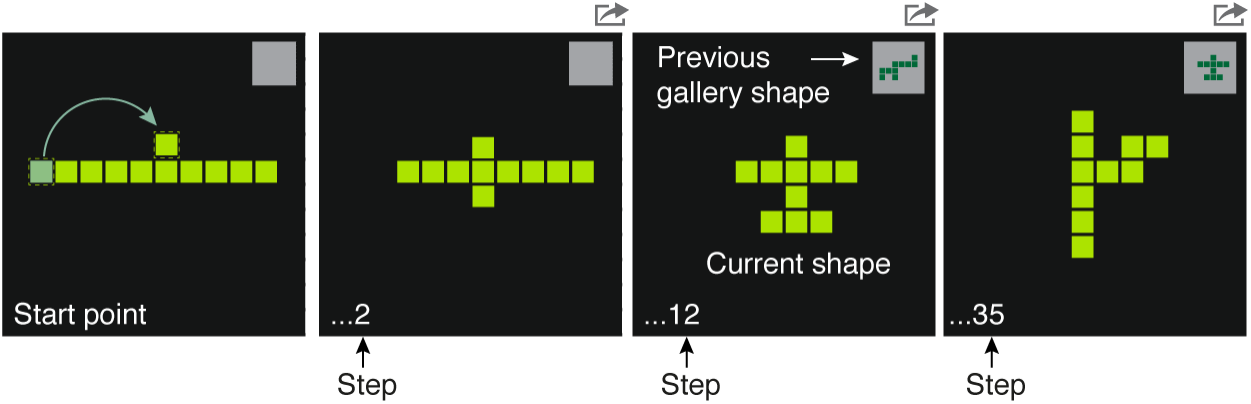
The creative foraging game allows high-resolution automated analysis of exploration and discovery processes. Players move squares starting from a 10-square line with the aim of finding ‘interesting and beautiful’ shapes. Moves keep all squares connected (not by a diagonal). A shape can be saved to a gallery by pressing the gray square at the top-right corner of the screen; in this square, the last shape chosen to the gallery is displayed (see Section B in S1 File).

The creative foraging game is a computer game in which players can move ten adjacent squares to create different shapes (Fig 1). There are 36,446 possible shapes (Section A in S1 File). The players are instructed to save shapes that they find interesting and beautiful into a gallery. The timing of all moves and gallery choices is recorded. This setting allows us to observe all intermediate steps as people search a large but finite metric space of different possibilities.

We present data on 100 participants and interpret the results in the context of OFT according to the following hypotheses:

(H_1_) Participants’ trajectories in shape space are composed of distinguishable phases of exploration and exploitation: exploration for new shape categories and exploitation of similar shapes within a category.

(H_2_) In phases of exploitation people move along optimal (shortest) paths.

Our third hypothesis diverges from the predictions of a naïve version of OFT, in which there is a constant value for any shape collected. The performance metric in naïve OFT is therefore the amount of collected resources. Hence naïve OFT predicts that exploitation of a given patch is terminated due to diminishing returns caused by depletion of the patch. In contrast, in creative foraging the intrinsic interest ‘value’ of collected resources is important. Collection from a given patch may lead to diminishing interest, far before the patch is depleted. We therefore hypothesize that:

(H_3_) In creative foraging participants leave exploitation phases sooner than predicted by naïve OFT, far before patch depletion.

The next hypothesis concerns the moment when a new category of shapes is discovered. We suggest that entry points to phases of exploitation can serve as an experimental proxy for creative leaps. Qualitative descriptions of creative processes suggest that creative leaps often occur in the periphery of a field [29], through a road that is not often taken. In addition, creativity was previously linked to ambiguity [30]. We therefore hypothesize that:

(H_4_) Shapes at the entry points to phases of exploitation are more ambiguous, in the sense that they can lead different players to different categories.

Finally, we hypothesize that

(H_5_) People show individual differences in their search strategies and patterns.

In the results section we operationalize these hypotheses in terms of the creative foraging task.

## Results

### Search dynamics are composed of alternating phases of exploration and exploitation

**Fig 2:**
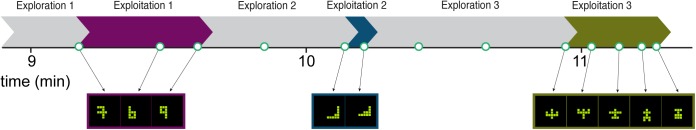
Search trajectories are segmented into exploration and exploitation phases. Segmentation uses the timing difference Δt between gallery shape choices. Periods with increasing Δt are labelled as exploration phases. An exploration phase ends with a decrease in Δt, leading to a sequence of rapid gallery choices, labelled as exploitation phases (see Methods, and Section C in S1 File). Choices of shapes to the gallery are marked with an open circle.

We analyzed games by 100 participants. Games lasted 15 min and averaged 306 steps (95% CI = [286,324]) and 46 shapes chosen to the gallery (95% CI = [41,50]). We analyzed the time series of gallery choices made by each player. The timing difference between subsequent gallery choices shows a distinct pattern: periods of slowing down where gallery choices happen more and more infrequently, and periods of acceleration where gallery choices occur more and more rapidly. We used a thresholding algorithm (Methods, and Section C in S1 File) that employs this timing data to segment the games into two alternating phases, which we name exploration and exploitation respectively (See Fig 2). Games showed a median of 7 exploration and exploitation phases (95% CI = [6,8]). Properties of the two phases are shown in Table 1.

**Table 1:** Characteristics of exploration and exploitation phases (Medians and 95% CI).

	Exploration		Exploitation		
	Median	95% CI	Median	95% CI	
Number of gallery shapes per phase	1.5	[1,1.5]	3	[3,3.5]	Exploration < Exploitation
Number of moves between gallery shape	9	[8,10]	4	[4,5]	Exploration > Exploitation
Duration of phase (sec)	53	[44,59]	50	[42,56]	Same
Number of moves per phase	16	[13,18]	17	[14,20]	Same
Duration of a single move (sec)	3	[2.9,3.4]	2.9	[2.7,3]	Same

### In exploitation phase players quickly harvest a sequence of similar shapes

**Fig 3:**
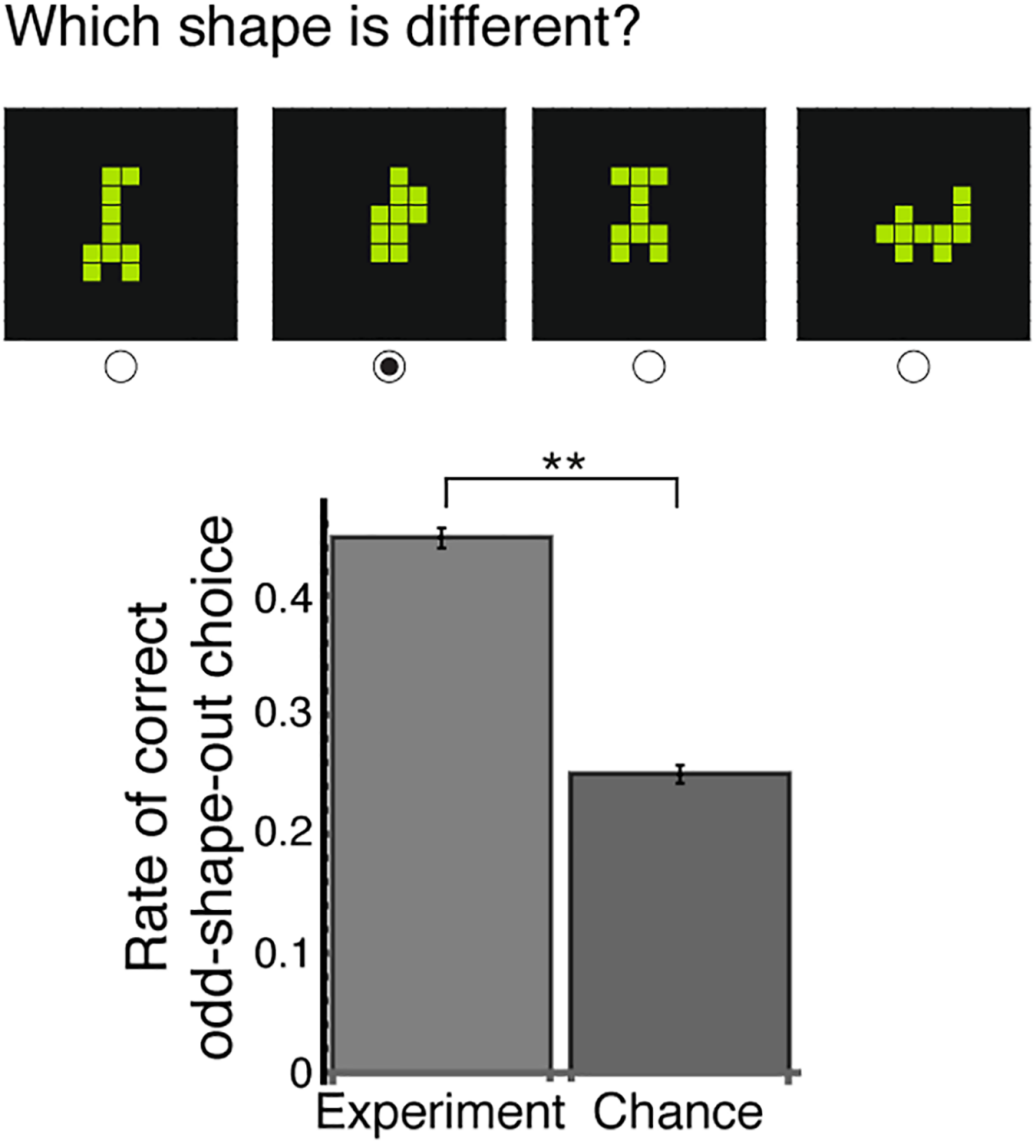
Shapes found in an exploitation phase are perceived as similar. Participants who did not play the creative foraging game chose the odd-one-out among four shapes, three from the same exploitation phase and one from a different exploitation phase found by the same player. Participants chose the outlier twice as often as by chance. Means and std (computed using bootstrapping) are shown (*p*<0.0001, see Section D in S1 File).

We noticed that the gallery shapes gathered in a given exploitation phase were similar to each other. To test this, we performed an ‘odd-shape-out’ test with a new set of participants who did not play the game (Fig 3). We find that people can differentiate between three gallery shapes taken from the same exploitation phase and a distractor gallery shape taken from a different exploitation phase (χ2(2, N = 67) = 1627, p < .0001, and Section D in S1 File). We conclude that players harvest a sequence of similar of shapes in each exploitation phase. They then leave this ‘patch’ of shapes to explore for new shapes (H_1_).

### Only in exploitation phases players move along direct paths between gallery shapes

Next, we considered a prediction of optimal foraging theory (OFT) that within exploitation phases, players move along direct (optimal, shortest) paths between discoveries [31]. To test this, we compared the length of players’ actual paths between two gallery shapes to the length of the shortest possible path between these shapes. In doing so, we took advantage of the fact that the current search space is well-defined and fully enumerated, so that the shortest path between any two shapes can be readily computed.

**Fig 4:**
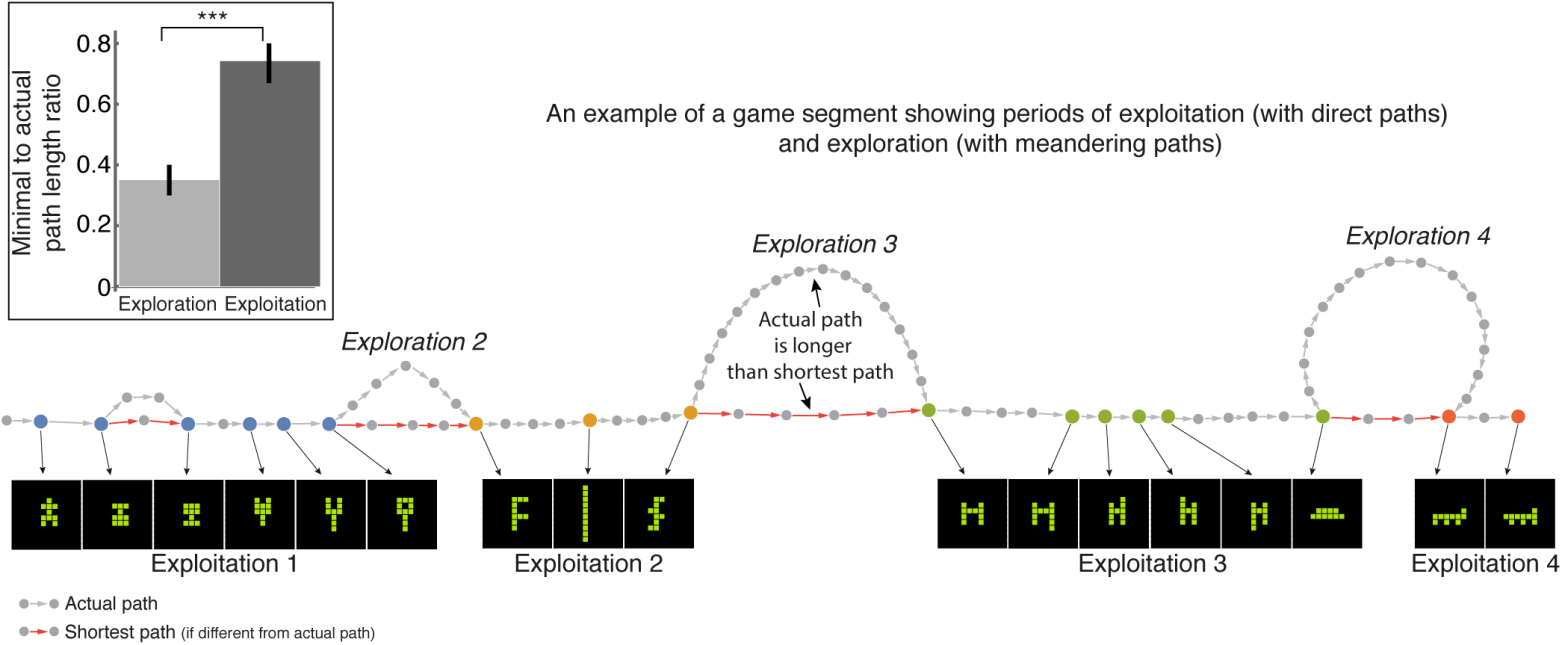
Participants follow optimal (shortest) paths in exploitation phases. Shown are moves (circles), gallery-chosen shapes (colored circles), actual (gray) and minimal (red) paths (minimal path marked when different from actual path). Exploration and exploitation phases are labelled. Inset, players’ median ratio of minimal to actual path length between gallery shapes in exploitation and exploration.

We find that during exploration phases players did not take the shortest path between gallery shapes; their paths averaged three times longer than the shortest possible path (Median shortest distance/actual distance = 0.35, 95% CI = [0.3,0.4]) (Fig 4). In contrast, in exploitation phases, players moved closer to the optimal path (Median shortest distance/actual distance = 0.74, 95% CI = [0.67,0.8], MW-test = 985, p < .001, effect = 0.8). These effects are evident also when comparing pairs of shapes with the same shortest distance in the two phases (Section E in S1 File). In summary, we find that in phases of exploitation–but not in phases of exploration—people move along optimal (shortest) paths (H_2_).

### Different players find similar shape categories

We noticed that different players rediscovered the same categories of shapes- for example numerical digits and shapes resembling airplanes. To study this, we developed an automated definition of shape categories. We defined a shape category as a set of shapes often rediscovered by different players. To do so, we used a network representation of shapes found by different players and used a community-finding algorithm to automatically detect shape categories (see Methods and Section F in S1 File). Each category includes all shared shapes found by different players during their game. Thus, categories aggregate shapes that were submitted by different players.

The algorithm revealed 14 categories that were found again and again by different players. These 14 shape categories contain a total of 653 shapes, 34% of all gallery shapes collected by participants.

**Fig 5:**
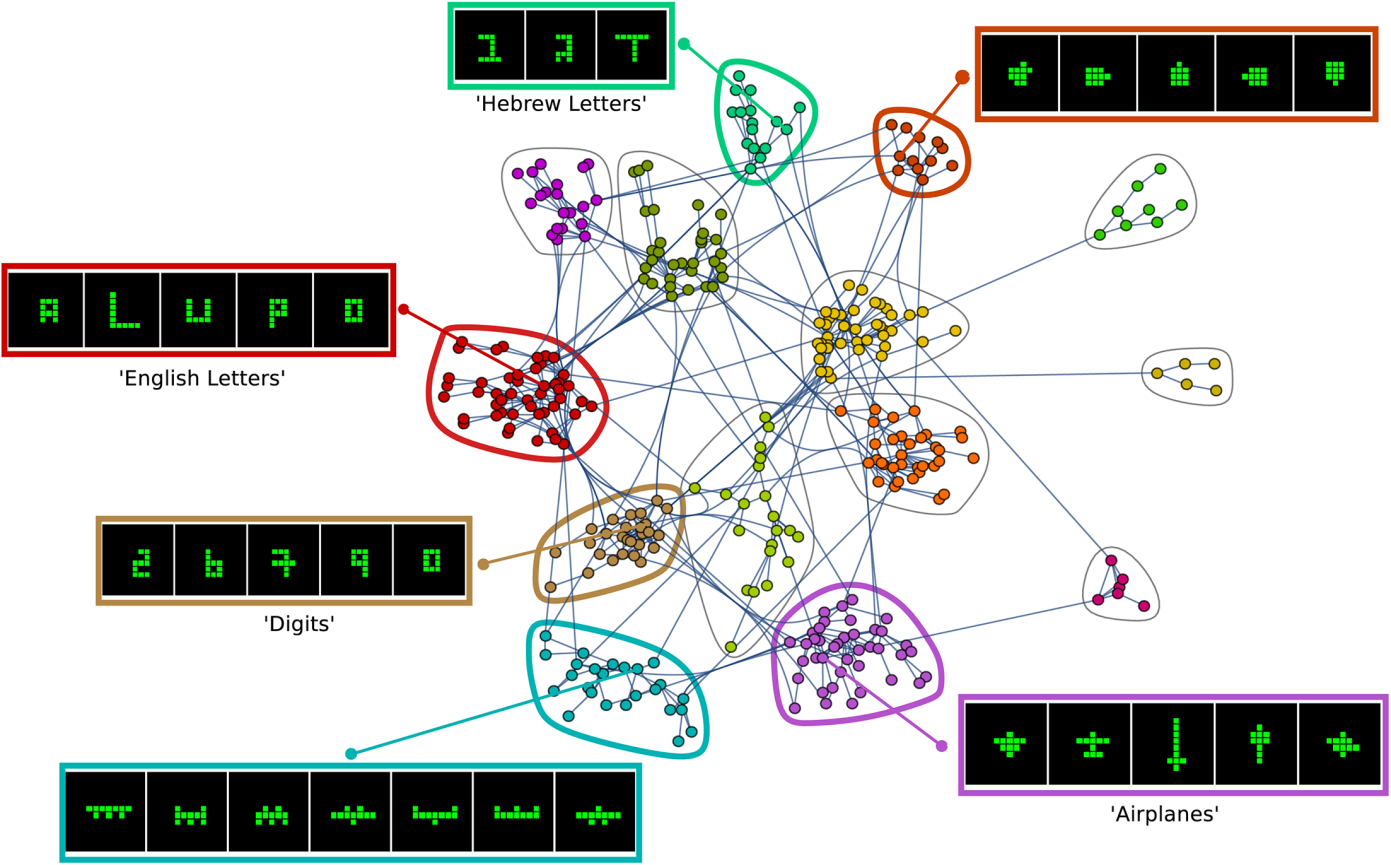
Different players discover similar categories of shapes in their exploitation phases. Network in which nodes are patches (shapes found in an exploitation phase) from 100 players. Links in the network connect patches that share at least two shapes. Categories are defined as Girvan–Newman modules in the graph, and are shown in colors, with representative shapes (see Section F in S1 File).

Each category includes shapes with a shared theme: familiar objects such as animals or airplanes, or symbols such as digits or letters. Other categories were quite abstract but seemed to share a visual similarity. Examples of shape categories are shown in Fig 5.

We tested whether people can reliably distinguish between these categories using an ‘odd-shape-out’ test, similar to the one reported above. We asked 86 people who did not play the game to match a randomly chosen triplet of shapes from a single category either to a set of six shapes from the same category or to a set of six shapes from another randomly chosen category (see Section G in S1 File). Participants chose the correct set 80±1% of the times, significantly more than chance (χ2(2, N = 86) = 1343, p < .001).

We conclude that the creative foraging game induces meaning on the space of shapes, with at least 14 different meaning categories. People explore until they hit upon one of these categories, then exploit that category, and then return to explore for new categories.

Interestingly, the shape categories are not separated from each other by many moves. Instead they are interleaved such that neighboring shapes belong to different categories (see Methods and Section H in S1 File). Players in an exploitation phase seem to focus their attention on one category, and pass by many shapes from other categories.

### Departure from exploitation phase is not due to depleted resources

Identifying the shape categories enabled us to test another prediction of optimal foraging. According to OFT, foragers leave a patch of resources when they begin to deplete it, a phenomenon known as diminishing returns. Diminishing returns are due to a decrease in the rate of harvesting resources. To test this we asked whether participants leave an exploitation phase when they are close or far from depleting the current category.

We find that players cover on average only 6.8% (95% CI = [6.1,7.4]) of the aggregated shapes of each category (see Section I in S1 File). Moreover, at the point of departure from an exploitation phase, the next potential shape belonging to the same category is very close, only 1.3 moves away on average (95% CI = [1.2,1.4]). Thus, players leave an exploitation phase far before the category is depleted (H_3_).

### Players showed individual differences along a continuum between fast and slow search strategies

We next asked about the difference in the search process of different people (H_5_). Players varied considerably in the average duration of their exploitation phases (Median = 50 sec; 5%-95% quantile range = [17,102] sec). We find that the duration of exploration and exploitation phases was positively correlated between participants: players with long exploration phases also had long exploitation phases (Spearman correlation, r = 0.80, 95% CI = [0.69,0.87], p<0.001).

**Fig 6:**
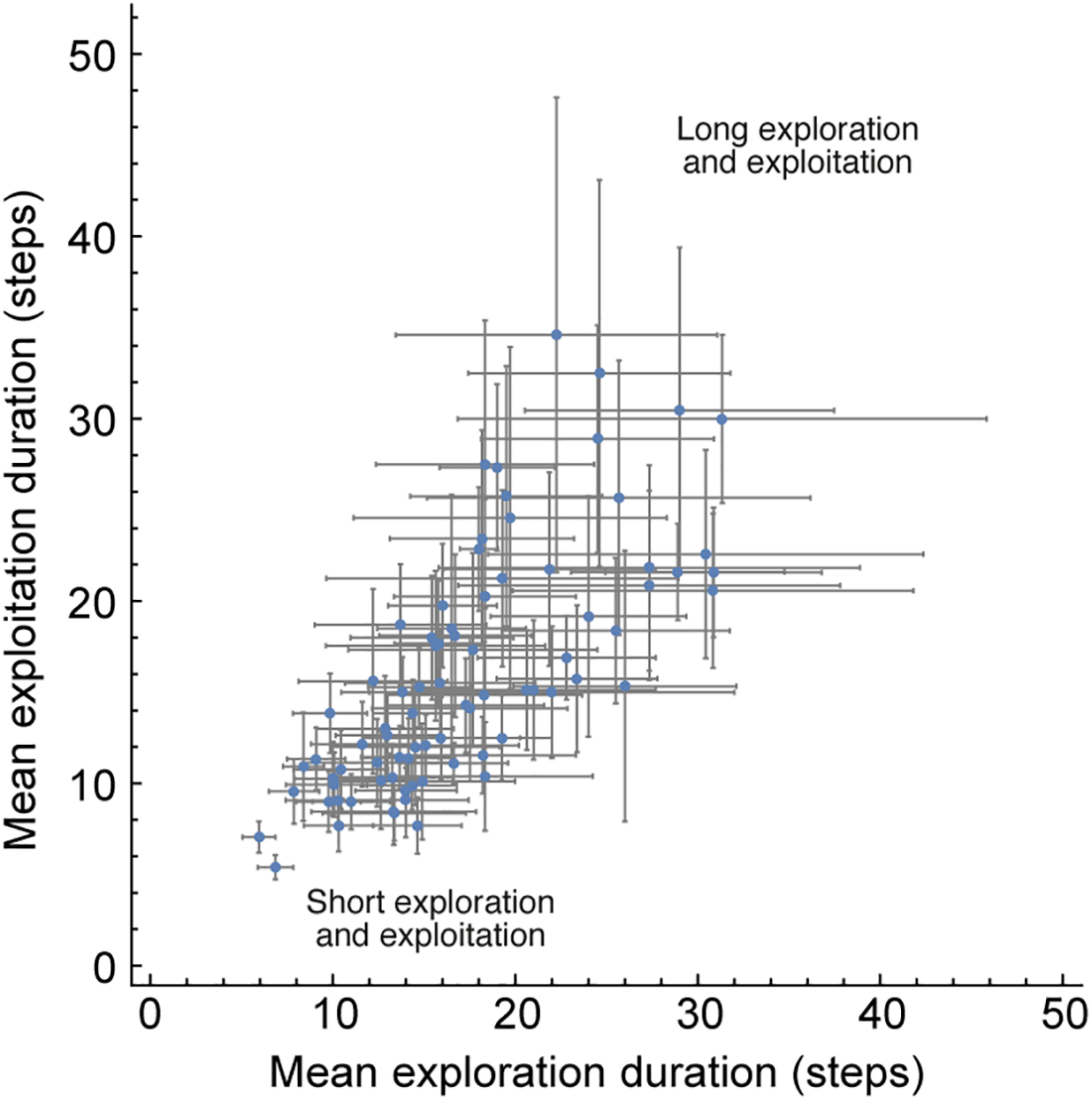
Players show correlated mean exploration and exploitation phase durations. Mean and error bars of one std are shown for each player. Spearman correlation, r = 0.78, 95% CI = [0.68,0.86], p<0.001.

To adjust for possible differences in individual move speeds, we plotted the mean number of moves in exploration and exploitation for each player (Fig 6). The average number of moves in exploration and exploitation phases also showed strong correlation (Spearman correlation, r = 0.78, 95% CI = [0.68,0.86], p<0.001). We controlled for the possibilities that these effects are the result of our segmentation algorithm or the general distributions of the duration of each phase (SI, Section J in S1 File).

We conclude that people in our sample vary along a continuum between two strategies: a fast strategy of short exploration/exploitation and a slow strategy of long exploration/exploitation. Those quick to discover are quick to drop, and those slow to discover are slow to drop and are thorough in their exploitation of a category.

### Entry into exploitation phases occur at ambiguous transition shapes at the periphery of shape categories

**Fig 7:**
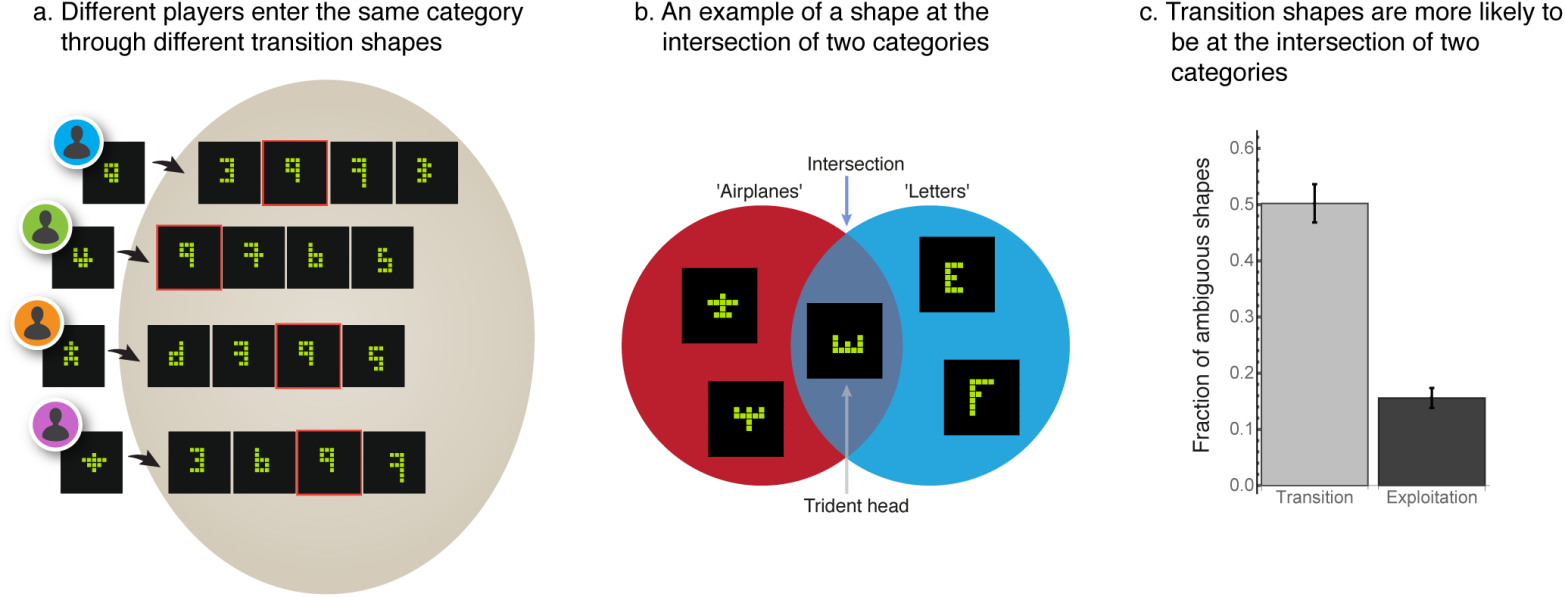
Discovery of a new exploitation phase occurs at a transition shape that is ambiguous in the sense that it belongs to multiple categories. **A)** Examples of transition shapes at the entry to the category of ‘digits’ by four different players. Transition shapes are non-prototypical, for example a ‘four that is not a four’, whereas shapes found after the transition shape tend to be more prototypical digits. **B)** A shape is ambiguous if it lies in the intersection of two or more categories (signifying at least two contexts of meaning), as exemplified by the trident shape shared by the categories of ‘airplanes’ and ‘English letters’. **C)** Transition shapes are more likely to be ambiguous shapes than non-transition gallery shapes (transition shapes: Median = 50%, 95% CI = [49, 50]; non-transition shapes: Median = 15, 95% CI = [15,16]).

The creative foraging game allowed us to focus on the moments of discovery of new categories of shapes. These moments occur at the transition between exploration and exploitation. We call the shape that marks the beginning of a new exploitation phase the ‘transition shape’. Different players enter exploitation of a given category through different transition shapes. For example, in Fig 7A, different players discover the ‘digits’ category through different transition shapes. One player entered the category of digits through a shape which we termed ‘the 9 which is not a 9’ (Fig 7A, first row), whereas another player entered the digits category through ‘the 4 which is not a 4’ (Fig 7A, second row). After finding the transition shape, players go on to find more prototypical digit shapes (Fig 7A).

Only 23% (95% CI = [20, 25]) of the players who found a transition shape use it as a start of a new exploitation phase; for other players who reached the same shape, it represents a ‘road not taken’ (Section K in S1 File). Of those that did go on to start an exploitation phase, the category exploited differs between players. In this sense, transition shapes are ambiguous (H_4_): they belong to multiple meaning categories more often than other gallery shapes (Fig 7B and 7C) (transition shapes: Median = 50%, 95% CI = [49, 50]; non-transition shapes: Median = 15%, 95% CI = [15,16], and Section L in S1 File).

### The creative foraging game can score creativity and correlates with the manual AUT

Finally, we compared the present test to a commonly used creativity test, the alternate uses test (AUT) [15]. The AUT provides two creativity measures: fluency- the total number of alternative usages found, and originality- the rarity of the solutions compared to a given dataset. We compared these measures to the corresponding measures from the creative foraging game, in a separate experiment on 57 people who played the creative foraging game (CFG) and took the AUT (Section M in S1 File). Fluency in the CFG was defined as the total number of gallery shapes found in the exploitation phase by the player; originality was defined by the frequency that the exploitation gallery shapes of the player were also found by the 100 players in our main dataset.

We find that both fluency and originality in AUT showed a trend in correlation with their respective measures in the CFG (N = 57, r = 0.18, p = 0.17 and r = 0.2, p = 0.14 for fluency and originality respectively). To assess the total matching between AUT and CFG, we defined a composite creativity score that averages over the two measures; we averaged the Z-transformed fluency and originality score of each player. We find that the composite creativity score in the CFG shows medium correlation with the composite creativity score in AUT (N = 57, r = 0.27, p = 0.04, CI = [0.1, 0.5]).

## Discussion

We presented the creative foraging game as a paradigm to study intrinsically motivated creative exploration. In the creative foraging game, people search a defined metric space of shapes for interesting and beautiful shapes. Multi-dimensional information about the players search trajectories is automatically recorded at high resolution.

We find that participants’ trajectories are composed of alternating phases of meandering exploration, punctuated by transitions to exploitation of patches of similar shapes (H_1_). Different people rediscover the same shape categories, including letters, digits, airplanes and other more abstract categories with shared visual properties. Thus, the creative foraging game induces meaning categories on the space of shapes. We find that these categories are interleaved rather than segregated in this space, such that shapes from a given category have neighbors from different categories. Hence, participants need only change their focus of attention in order to discover a new category. This contrasts with a common metaphor in which creative search is like seeking an oasis of novel and valuable solutions in the desert of ordinary. Search in an interleaved landscape might be relevant for exploring creative solutions in information-rich digital environments.

The existence of exploration and exploitation phases suggests a link to studies on human foraging behavior. Human foraging studies supported the predictions of OFT [18,20]. In line with OFT, we find that in exploitation—but not in exploration—participants follow the optimal path (shortest possible path) between the collected shapes (H_2_). This finding of meandering paths in exploration versus direct paths in exploitation can ground the metaphor from creativity research of ‘fogginess’ in exploration vs. ‘visibility’ in exploitation [27,32].

Our results differ from OFT regarding the point of departure from an exploitation phase, highlighting the different nature of the goals of naïve OFT and creative foraging. Naïve OFT predicts that foragers leave a patch due to diminishing returns caused by resource depletion. In contrast, we find that participants exit exploitation far before their patch of shapes is depleted (H_3_). This difference might be the result of the goals of naïve OFT and creative foraging. OFT assumes that resources have a defined and time-invariant value, and hence foraging aims to maximize the amount of discovered solutions, where all solutions have the same quality. In contrast, in creative foraging the intrinsically evaluated ‘quality’ of solutions varies considerably—some solutions are novel and interesting while others are more dull. The process of evaluating solution quality may be dynamic: the value of the first exemplar from a category is higher than the value of the nth exemplar, suggesting that creative foraging is guided by a mechanism related to novelty seeking [33,34]. Finally, the present experimental design had no explicit incentive to deplete categories of shapes, or to find as many shapes as possible. Such incentives may alter the observed behavior.

We also studied individual differences in search strategies. We find strong correlation between the lengths of exploration and exploitation phases: while some people tend to explore for a short time and exploit for a short time (a mercurial strategy of quick to find, quick to drop), other explore longer and exploit longer (a thorough strategy of slow to find, slow to drop). This correlation is preserved also when we adjusted for the speed in which people make the moves in the game. Other possible strategies such as short exploration and long exploitation (quick to discover and thorough exploitation) seem to be missing in our sample.

The quick-to-discover/quick-to-drop versus the slow-to-discover/slow-to-drop behavioral continuum may hint that a common mechanism with a single time-scale governs both exploration and exploitation phases in the creative search process. A similar connection between the two phases was shown in previous studies. For example, in decisions from experience-sampling paradigms, people who switch more rapidly between options tend to sample less before making a decision [35]. Similarly, in a study based on a binary prediction task, earlier exploitation behavior correlates with more exploration [36]. Hills and colleagues [21] explain these behaviors as resulting from an executive search process model that is ruled by a single time scale, the updating parameter. Faster updating means that 'distractors' more easily influence exploitative phases. In turn, this leads to a low threshold for exploitation and a low threshold for exploration.

More subtle differences in exploration and exploitation strategies between individuals might be observed with a larger pool of participants. Future research can explore possible mechanisms in terms of different personality traits [37] or attention deficits [38], and to link these findings with the changes in brain activation that have been associated with exploration and exploitation states [39].

Finally, we characterized the moments of discovery of new categories using the shapes at which participants enter a new exploitation phase. We find that the transition between exploration to exploitation occurs through shapes that are more ambiguous (belong to more than one category, H_4_) than other gallery shapes. We suggest that these transition shapes can serve as an experimental proxy for moments of insight [40].

It is encouraging that the present test correlates reasonably well with arguably the most commonly-used manual test for creativity, the AUT. We note that the creative foraging game is a search task on a structured and bounded space of solutions, compared with the much more open-ended AUT. As such, it might lack the strong correlations between fluency and originality exhibited in the AUT test, but provides a possible automated and scalable creativity measure for further studies to explore.

The present study has several limitations: first, the participant pool is relatively small and from a single culture. This limitation can be readily overcome in future studies due to the suitability of the creative foraging game for online platforms. Second, participant creativity is impacted by the limited space of geometric shapes we ask them to explore. However, the effect of limitations on creative exploration is not clear, and some research suggests that certain constraints can enhance creativity [24,41]. Finally, our paradigm addresses only a thin slice of creative exploration [42], and can be categorized as a study on small-c creativity [43].

Keeping these limitations in mind, the present paradigm can boost creative exploration research by providing automated information on how people explore, including intermediate steps, in a situation where solutions can be compared and their distance computed. The creative foraging game may thus open a window to study mechanisms, traits and interventions that can improve creative search.

## Methods

### Participants

Undergraduate students at the Hebrew university (54 females, 46 males, age 20–49, mean(±std) = 25(±4)), took part in the experiment either for credit or payment equivalent to 5$. This study was approved by the IRB committee of the Hebrew University of Jerusalem.

### Creative foraging game

The creative foraging game was run on a PC with on-screen instructions. Players created shapes by moving at each step one of ten identical squares, keeping the squares connected by an edge (and not a corner, see Fig A in S1 File). The initial condition was ten squares in a horizontal line. Participants were given the following instructions: *“Your goal is to explore the world of shifting shapes and discover those that you consider interesting and beautiful”*. At each point in the game players could store the current shape to a gallery by pressing a gray square at the top-right side of the screen. The gray square showed the last gallery shape chosen. The gallery had no limit on the number of shapes. Games lasted 15 minutes. After the game, players performed another task—choosing the five most creative shapes from their gallery. This task is not analyzed in the current study.

### Links to the creative foraging game and data

The game can be accessed at: http://www.weizmann.ac.il/mcb/UriAlon1/Cubes/welcome.html

The raw data used in the analysis described in the main text can be accessed at: http://www.weizmann.ac.il/mcb/UriAlon/download/downloadable-data

and at: https://figshare.com/articles/Online_data_for_Creative_Foraging_paper/5162149

### Segmentation algorithm

A segmentation algorithm defined the exploitation and exploration phases in each game. The input is the series of timing difference in seconds between consecutive choices of gallery shapes. The output is a labeling of gallery shapes into exploration and exploitation phases (Fig B in S1 File). The algorithm has two iterations. In the first iteration, consecutive shapes are grouped together into an exploitation phase if their timing differences monotonically decrease. The second iteration groups together consecutive exploitation groups from the first iteration if the maximal timing difference in the earlier group is larger than the maximal time difference in the second group. The purpose of the second iteration is to avoid fragmentation of exploitation phases due to a single large time-difference value (Section C in S1 File for further details). Shapes that remain outside of exploitation phases are labeled as exploration phases.

### Odd-shape-out test of perceived shape similarity

We tested the similarity of shapes within the same exploitation phase by means of an odd-one-out test. Participants who did not play the creative foraging game (31 males, 36 females; age range: 20–80, mean (±std) = 36 (±12)) observed one of four blocks of 50 quartets of shapes. Sample size was calculated assuming medium effect size, and was sufficient for statistical power exceeding 0.95 assuming μ_0_ = 0.25, μ_1_ = 0.4, σ = 0.05, α = 0.05. Each quartet included three shapes from the same exploitation phase and a fourth shape from a different exploitation phase found by the same player, in randomized order (see Fig 3). A total of 200 randomly selected exploitation phases were tested out of 791 in the data-set (~25%). The experiment was performed on a commercial platform for online surveys (Qualtrics, see: http://qualtrics.com/). Error bars were computed by bootstrapping 10,000 times. See Section D in S1 File for further details.

### Shape categories

We defined categories of shapes found by different players in exploitation phases using a network community approach. We constructed an undirected network in which each node is a group of shapes found in a single exploitation phase, hereafter termed patch. Two patches were connected in the network if they share at least two shapes. The network had a giant component of 334 patches and 17 smaller connected components (containing less than 8 patches each with a total of 46). We defined shape categories by finding communities (modules) in the giant component using the Girvan–Newman algorithm. We find 14 shape categories of varying sizes (mean number of shapes (±std) = 70 (±38)). See Table A in S1 File for the characteristics of the shape categories and Fig E in S1 File for all the unique shapes in the 4 biggest categories.

### Perceptual test of within-category similarity

We tested the perceptual grouping of the categories by a grouping experiment. 86 participants who did not play the game (43 males, 43 females; age range: 18–77, mean (±std) = 41.6 (±15.2)) were asked to match a triplet of randomly chosen shapes from the same category to one of two groups–six random shapes from the same category or six random shapes from another randomly chosen category (see Fig E in S1 File). Sample size was calculated assuming medium effect size, and was sufficient for statistical power exceeding 0.95 assuming μ_0_ = 0.5, μ_1_ = 0.7, σ = 0.05, α = 0.05. We created for each of the largest nine categories a set of 40 such questions for a total of 360 unique questions. Groups of at least 10 participants responded to one of eight blocks of 45 questions posed in random order. See Section G in S1 File for further details.

### Interleaved meaning categories

To test whether meaning categories are separated or interleaved, we computed the shortest path between all pairs of gallery shapes within a category, and between all pairs in different categories (total of N = 653 shapes yielding ~212K pairs). See Section H in S1 File for further details. We find that the two distributions of path lengths are similar, suggesting an interleaved geometry (between categories: Median = 4 steps, 95% CI = [4,4], within categories: Median = 4 steps, 95% CI = [4,4], effect = 0.06, see Section H in S1 File). Second, we enumerated the number of potential gallery shapes that could be reached in K steps from a given gallery shape S. We computed the ratio between potential shapes that belong to the same category as S, and potential shapes that belong to all other categories. We find that the probability of choosing a next gallery shape that belongs to the same category, for a given number of steps, is about 1:4 (Median = 0.25, 95% CI = [0.24,0.26], see Section H in S1 File). We conclude that players do not stay within the same category in a given exploitation phase only because of the proximity of these shapes.

### Comparing AUT and CFG scores

In creativity tests such as AUT and TTCT, experimenters have access to the submitted solutions provided by the participants verbally or in drawn form, but not to intermediate steps or solutions. These submitted solutions carry a clear conceptual meaning, which in the CFG corresponds to the gallery shapes found in exploitation phases. We therefore scored exploitation gallery shapes rather than all gallery shapes in CFG. We note that considering all gallery shapes slightly improves all correlations: fluency (CFG)-fluency (AUT)- r = 0.2, p = 0.13, originality (CFG)-originality (AUT)- r = 0.21, p = 0.12, and the correlation of the CFG and AUT composite scores is- r = 0.29, p = 0.02.

## Supporting information

S1 File Supporting information file. Supporting Information for methods and data analysis.
(DOCX)
