# Supplementary material for: Creative foraging: An experimental paradigm for studying exploration and discovery
Source: PLoS One. 2017 Aug 2;12(8):e0182133. doi: 10.1371/journal.pone.0182133 (PMC5540595; doi:10.1371/journal.pone.0182133)
Supplement: S1 File — Supporting Information for methods and data analysis. (DOCX) [file pone.0182133.s001.docx]

**Supplementary Information

Creative Foraging: an Experimental Paradigm for Studying Exploration and Discovery**

Yuval Hart^1,2,3^, Avraham E Mayo^1,2^, Ruth Mayo^4^, Liron Rozenkrantz^1,5^, Avichai Tendler^1,2^, Uri Alon^1,2*^ and Lior Noy^1,2*^

^1^ The Theatre Lab, Weizmann Institute of Science, Rehovot, Israel

^2^ Department of Molecular Cell Biology, Weizmann Institute of Science, Rehovot, Israel

^3^ Paulson School of Engineering and Applied Sciences, Harvard University, Cambridge, MA, USA

^4^ Department of Psychology, The Hebrew University of Jerusalem, Jerusalem, Israel

^5^ Department of Neurobiology, Weizmann Institute of Science, Rehovot, Israel

* Corresponding authors: [uri.alon@weizmann.ac.il](mailto:uri.alon@weizmann.ac.il) (UA) and [lior.noy@weizmann.ac.il](mailto:lior.noy@weizmann.ac.il) (LN)

Table of Contents

[A) The structure of the shape space 3](#_Toc488012062)

[B) Setup of the main experiment: further information 3](#_Toc488012063)

[C) The exploration/exploitation periods segmentation algorithm 4](#_Toc488012064)

[D) ‘Odd-shape-out’ test: further information 4](#_Toc488012065)

[E) Shape pairs with the same minimal path between them have different actual path length in the exploitation and exploration phases 5](#_Toc488012066)

[F) Patches from different players can be grouped to categories with common themes 6](#_Toc488012067)

[G) Grouping test shapes in the same category: further information 6](#_Toc488012068)

[H) Categories are interleaved in shape space 7](#_Toc488012069)

[I) Players leave a category before it is depleted 8](#_Toc488012070)

[J) Players’ exploration-exploitation durations are correlated independent of the segmentation algorithm and timing distributions 8](#_Toc488012071)

[K) Players often pass through possible transition shapes without entering new categories 8](#_Toc488012072)

[L) Examples of transition shapes to multiple categories 9](#_Toc488012073)

[M) Comparing the current paradigm to common tests of creative thinking 9](#_Toc488012074)

[References 10](#_Toc488012075)

[Figures 11](#_Toc488012076)

[Tables 21](#_Toc488012077)

[Appendix – complete written instructions for the experiment. 22](#_Toc488012078)

# A) The structure of the shape space

The underlying structure of the current study is the space of all shapes composed of 10 connected squares (diagonals do not count as connections, so that each square in a shape has up to 4 neighbors). We enumerated all 36,446 shapes in the space. We counted each shape as distinct, but note for completeness that these shapes can be generated from 4,655 shapes using rotation and reflection symmetry.

We also analyzed the structure of the network of shapes reached by the human participants. We constructed a directed graph with a vertex for each shape reached by at least one player, and an edge for each move between two shapes. The 100 participants covered 10,789 (30%) of the shapes in the space. Participants covered only a small fraction of the possible moves (24,584 edges, 2% of possible edges), which is not surprising considering the large amount of possible moves in the space (~10^6^).

# B) Setup of the main experiment: further information

**Participants**

One hundred students at a large university (54 females, age 20-49, mean age (±std) = 24.9(±3.5)), took part in the experiment either for credit or payment equivalent to 5$. The creative foraging game was presented to the participants as one of three separate experimental tasks. The game was a filler task between two tasks included in a different study.

**Method**

The creative foraging game was run on a PC. Written instructions appeared on the screen (see complete instructions in the Appendix). The game had two stages. In the first stage, players created shapes by moving one of ten identical squares at a time. Allowed moves kept the shape connected at the edges (not in the diagonal, see Fig A). The shape was kept in the center of the screen. The starting point for the search was the horizontal line. Players were given the following instruction:

*“Your goal is to explore the world of shifting shapes and discover those you consider as interesting and beautiful.”*

At each point in the game players could store the current shape to a gallery by pressing a gray square at the top-right side of the screen. The gray square showed the last gallery shape chosen. The full gallery was not visible during this stage. After 15 minutes, and after saving at least 5 gallery shapes, players moved to the second stage by pressing a small red button at the lower-right corner of the screen. They were then asked to choose among the gallery shapes:

*“Please choose the 5 most creative shapes you discovered.”*

They were then presented with the full gallery, chose five shapes, and clicked a similar red button to finish the game (see Fig A).

**Links to the game and the data**

The game can be accessed at: <http://www.weizmann.ac.il/mcb/UriAlon1/Cubes/welcome.html>

Link to raw-data used in the analysis described in the main text can be accessed at: <http://www.weizmann.ac.il/mcb/UriAlon/download/downloadable-data>

and at: <https://figshare.com/articles/Online_data_for_Creative_Foraging_paper/5162149>

# C) The exploration/exploitation periods segmentation algorithm

In this section we describe the algorithm for defining the two states in each game – exploitation and exploration. The input is the series of time differences in seconds between gallery choices. The output is a labelling of groups of consecutive gallery shapes as patches - each patch corresponding to one exploitation phase. The remaining shapes, not in patches, are labelled as exploration phases (Fig B).

The algorithm has two iterations. In the first iteration, shapes are grouped together if the timing differences are monotonically decreasing. For example, if the series of time differences between gallery choices is A = (10 20 70 30 20 40 90 40 10), the algorithm groups together the two underlined sets- each of which corresponds to an exploitation phase. The non-underlined values are exploration phases.

The second iteration groups together adjacent exploitation phases if the maximal timing difference in the first is larger than the maximal time difference in the second. In the example A above, this second iteration would not join the two phases, because there is an intervening exploration phase. An example where exploitation phases are joined is B = (10 20 70 30 20 50 40 10). The first iteration would partition B with two consecutive phases B1 = (10 20 70 30 20 50 40 10). The second iteration would then join the two phases, because 70 is larger than 50: B2 = (10 20 70 30 20 50 40 10).

The purpose of the second iteration is to avoid fragmentation of patches due to a single large time-difference value.

# D) ‘Odd-shape-out’ test: further information

The experiment was performed on a commercial platform for conducting online surveys (Qualtrics, see: <http://qualtrics.com/>). Participants were recruited by email asking to ‘participate in a short experiment in shape perception, as part of an on-going study’. Participants signed a consent form, entered basic demographic data (age, gender and dominant hand) and were presented with the following instructions:

*In the following experiment you will be presented with groups of four simple shapes. For each group we will ask you to mark one of the four shapes that you feel is different from the rest. To choose, please click on the circle under the shape you feel is different from the rest. There are 50 questions, and the experiments should take 7-8 minutes.
Thanks for taking part in this study!*

The Fleiss’ Kappa coefficients of the four sets were (0.44, 0.32, 0.38, 0.4), indicating ‘Fair’ to ‘Moderate’ interrater agreement (all Z > 42.4, all *p* < 10^-4^). Summing over all participants and items, we counted the number correct-choice of the odd-shape-out, and compared it to a random selection. Participants chose the correct odd-shape-out 45±1% of the times (error computed by bootstrapping the response vector 10,000 times), compared with chance choice of 25±0.6%, see Fig 3 in the main text, and Fig C (*χ^2^(2, N=67)* = 1627, *p* < 10^-4^).

We also replicated the current experiment in a different on-line survey platform (Panel4All, <http://www.panel4all.co.il/>). 94 participants (45 males, 49 females; age range: 18-74, mean age (±std) = 36.2(±11.3)) were asked to participate in the experiment. Participants were compensated (~2$). The results were almost identical to the results reported above, with a correct ‘odd-shape-out’ ratio of 43±1% (with std computed as above), compared with chance choice of 25±0.6%, and the observed correct odd-out ratio statistically different from the expected by-chance ratio (*χ^2^ (2, N=4700)* = 2499, *p*<10^-4^).

Combining each of the four 50-questions sets from the two experiments, and computing the Fleiss’ Kappa coefficients resulted in similar, slightly lower, values (0.42, 0.27, 0.33, 0.37), indicating ‘Fair’ to ‘Moderate’ agreement.

Considering the response pattern in each experiment as a vector of 800 numbers (200 questions * ratio of choosing for each of the four answers), we find that the two vectors are highly correlated (Pearson’s correlation, *r* = 0.9, *p* < 10^-4^). Thus, the two datasets show almost identical results.

# E) Shape pairs with the same minimal path between them have different actual path length in the exploitation and exploration phases

The path length between two gallery shapes in the exploitation phase are closer to the minimal (shortest) possible path than in the exploration phase, as described in the main text. We verify here that this difference is not the result of the differences between the distributions of the minimal path in the two phases.

To do so we computed the ratio of minimal-to-actual path for pairs of shapes with the same minimal path (Fig D). We conclude that paths in exploitation phases have a lower minimal/actual ratio also when controlling for the minimal path length between the shapes.

# F) Patches from different players can be grouped to categories with common themes

Here we detail our approach to define categories of shapes. We constructed an undirected network of connections between patches (groups of gallery shapes found in an exploitation phase) based on the rule that two patches by two different players are connected if they share at least two shapes. This procedure yields a network made of one giant component with 334 patches and 17 smaller components (containing less than 8 patches each, with a total of 46 patches). Hereafter we concentrate on the giant component.

In order to find the categories, we use the Girvan–Newman algorithm for finding modules in the given network (1). A module is defined as a sub-network which contains more intra-connections compared to inter-connections to other modules. We find 14 modules with varying sizes (mean number of shapes (±std) = 70 (±38)). We consider each module as a category of shapes with a common theme. Table S1 presents statistics of the categories. To demonstrate the shapes composing a shape category we present in Fig E all the unique shapes in the four biggest categories.

Many patches are rediscovered by other players - we find that 48% of the patches share at least two shapes with another patch, and 98% of the patches share at least one shape with another patch. We show in Fig F panel C the histogram of the number of connections per patch, a measure that indicates the number of times a patch was rediscovered by other players. We find that 61% of the patches in the giant component were rediscovered by four players or more.

# G) Grouping test shapes in the same category: further information

The experiment was conducted on a commercially available platform for on-line experiments (Panel4All, see: <http://www.panel4all.co.il/>). Participants were recruited among Hebrew-speaking people who are registered to this platform, and were paid the equivalent of 2$ for their participation.

86 participants (50% females; age range: 18-77, mean (±std) = 42 (±15)), who did not participate in the other experiments, responded to a set of 45 questions (out of 8 sets; total of 360 unique questions) in randomized order. Each question presented a triplet of shapes from one category and two possible matches: a group of six shapes from the same category, and a group of six shapes from a different category (see Fig E).

Participants were asked to choose - by clicking on one of the two groups of six shapes - the one which is more similar to the group of three shapes.

40 different questions were created for each of the nine biggest categories. Questions were created by randomly choosing 3 shapes from that category for the triplet, randomly choosing 6 shapes from the same category for the correct response and randomly choosing one of the other eight categories, and randomly choosing shapes from that category for the distractor. This produced a total of 9*40=360 unique questions, and each question was presented to at least 10 participants.

The experiment took ~10 minutes and was self-paced. Participants signed a consent form, provided demographic data and read the instructions for the experiment. We find that participates chose the correct category 80±1% of the times (error computed by bootstrapping the response vector 10,000 times), compared with chance choice of 50±1% (χ2(2, N=86) = 1343, *p* < 10^-5^).

There was some variability between different categories, with the most recognized category with 87% success, and the least recognized category with 67% (see Table S2), and std of 7% between categories. The high correct response rate was not limited to the three categories with clear semantic labels (C1, C2 and C7).

# H) Categories are interleaved in shape space

In this section we describe the procedure we used to test whether shapes within a category are clustered together because they are closer in shape space than they are to other shapes. To do so, we calculated all distances between shapes within each category and between each shape in a category and all shapes in other categories. We find that for both distributions the median distance between a pair of shapes is 4 moves, with 95% CI=[4,4] (See Fig G). We conclude that shapes in a category are not clustered together in shape space.

In order to further test the clustering of shapes we considered all shapes at a given distance (number of moves) from a shape and calculated the fraction of shapes that belong to the same category, and the fraction that belong to other categories. We find that for every shape that belongs to the same category there are on average 3 other equally distanced shapes which belong to other categories (see Fig G for the full distribution). Thus, categories with shared meaning do not seem to be concentrated in limited regions of shape space. Instead, they seem to be interleaved in shape space, such that each shape in a category has many close-by shapes from other categories.

# I) Players leave a category before it is depleted

For each player in each exploitation phase we calculated the ratio of shapes they collected versus the total number of shapes in the category. As shown in Fig H the median across different players for different categories does not exceed 10% and their mean is 6.8%. We conclude that players exit an exploitation phase well before depleting the shapes in the category.

# J) Players’ exploration-exploitation durations are correlated independent of the segmentation algorithm and timing distributions

In the main text we show that players’ mean duration of exploration and exploitation phases are highly correlated. Here we check that this correlation doesn’t stem from the way we segment the shapes nor from the timing distributions of each phase.

In order to control for the segmentation process and timing distributions, we use bootstrapping (1000 times) where in each run we produce 100 games, randomly sampling from the distribution of timing differences between shapes. We then apply the segmentation algorithm for each game and calculate the correlation between exploration and exploitation durations, we repeat this process 1000 times. Fig I shows that the randomized games distribution does not overlap with the correlation we find in the real data, suggesting that the segmentation algorithm and the timing distributions themselves are not a dominant driver of the correlation (p < 0.001).

# K) Players often pass through possible transition shapes without entering new categories

We define a transition shape as the entry shape into a new patch. We wanted to check whether a transition shape to a given category is common to all players or whether each player has a different transition shape. While there are few common shapes that when encountered, players switch to a particular category, most transition shapes are not shared by other players. In Fig J we show a histogram of the frequency of shapes being a transition shape. We find that 77% of the transition shapes are unique to a specific player.

In order to test for the idiosyncratic nature of each transition shape, we calculated the ratio between the number of times a shape was transitional and compared with the number of times a shape was found by different players. We focus the analysis on shapes found more than 4 times, since low-frequency shapes will have high ratios just because of their limited exposure. We find that only 23% of players (95% CI=[0.2,0.26]) reaching a transition shape actually use this shape as transitional and enter an exploitation phase. In Fig J we show the distribution of this ratio across all transition shapes.

# L) Examples of transition shapes to multiple categories

While most transitional shapes are unique to a player, there are a few shapes that are shared by several players. Moreover, some shapes served as transition shapes to different categories for different players. For example, a ‘trident head’ shape (an E shape rotated 90° counter-clockwise) may serve as an entry point to a patch of Latin letters (resembling the letter ‘E’), to a patch of Hebrew letters (resembling the Hebrew letter ‘Shin’ (\ש), or to a sequence of ‘airplane’-like shapes (see Fig J).

# M) Comparing the current paradigm to common tests of creative thinking

We compared the creative foraging game (CFG) to two manual tests of divergent thinking, commonly used to assess creative thinking (2) - the Alternative Uses Test (AUT) (3); and one part (Figural part, activity 3 (circles)) of the Torrance Tests of Creative Thinking (TTCT) (4). This comparison was part of a larger study on a placebo effect for creativity.

57 participants, who did not participate in the main study, took part in the experiment (49% females; age (mean±std) = 26 (±2.8)). All participants were a-priori and pseudo-randomly assigned into two groups: the placebo group (N=27) and the control group (N=30). There were no significant age and gender differences between the two groups.

All participants were presented with an odorant in a jar (cinnamaldehyde, CAS 104-55-2) and were asked to smell it, and to rate its intensity, pleasantness and familiarity. The placebo group was also told that the odorant is *“a unique odor, developed in our lab, which is designed to increase creativity and lower inhibitions”.*

Participants then played the CFG for 10 minutes. They smelled and rated the odorant again, and then completed the AUT and TTCT (each for 10 min) in a counterbalanced order.

The AUT and TTCT were manually coded for fluency (number of solutions) and uniqueness (the inverse of the frequency of the response in our dataset) following standard practice. For the CFG, fluency and originality can be defined either on the exploitation shapes or on all gallery shapes. In the main text, we report the correlation results on exploitation gallery shapes. Here, we report on the same calculation when analyzing all gallery shapes.

The correlation between the individual scores of fluency and originality between the tests showed medium-low correlation with a trend: fluency (CFG)-fluency (AUT)- N=57, r=0.2, p=0.13 and originality (CFG)-originality (AUT)- r=0.21, p=0.12. TTCT individual scores did not correlate significantly with either CFG or AUT. In all three tests, fluency and originality were correlated (CFG: Spearman correlation, r = 0.29, p=0.03; AUT: r = 0.81, p < 0.001; TTCT: r = 0.42, p = 0.001). We defined a composite score in all three tests. For each test, we Z-transformed uniqueness and fluency and then added these two Z-scores to arrive at a composite score for each player. The composite scores were correlated between the CFG and AUT (Spearman correlation, r = 0.3, p=0.02). The composite scores were not correlated between CFG and TTCT (r = -0.001, N.S) or between AUT and TTCT (r=0.11, N.S).

# References

1. Girvan M, Newman MEJ. Community structure in social and biological networks. Proc Natl Acad Sci. 2002 Jun;99(12):7821–6.

2. Plucker JA, Makel MC. Assessment of creativity. In: The Cambridge handbook of creativity. 2010. p. 48–73.

3. Guilford JP, Christensen PR, Merrifield PR, Wilson RC. Alternate uses: Manual of instructions and interpretation. Orange; CA: Sheridan Psychological Services.; 1978.

4. Torrance EP. Torrance Tests of Creative Thinking: Norms-technical Manual. Research Edition. Verbal Tests, Forms A and B. Figural Tests, Forms A and B. Personell Press; 1966.

# Figures


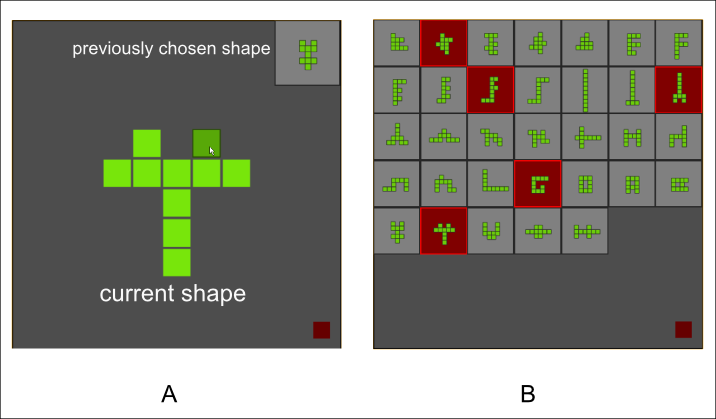


**Figure A:** **Screen shots of the creative foraging game.** **A)** During the game players create new shapes by moving a square (the active square marked in darker green) to a new location, while keeping the ten squares connected (by full edges, not diagonals). Players are asked to search for shapes that they find ‘interesting and beautiful’. They can save the current shape to an offline gallery of shapes by pressing the gray square at the top-right corner. During a move, the previously chosen shape is shown at the top-right corner. The full gallery is not visible during the search. Players end the search-and-save phase after 15 minutes, by pressing the red bottom at the lower-right corner. **B)** After finishing the first stage, the players are presented with the full gallery and are asked to choose ‘the five most creative shapes you discovered’ (marked in red).


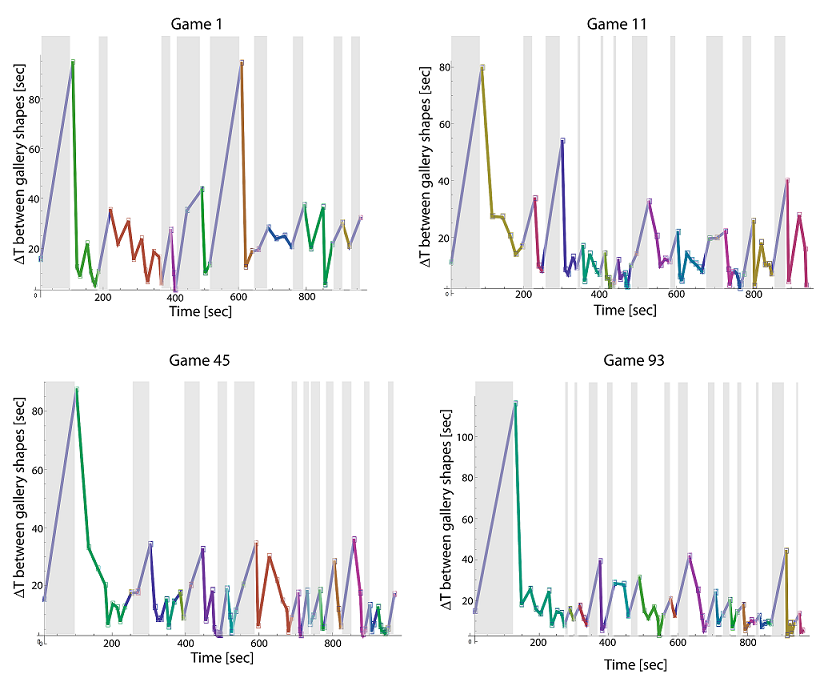


**Figure B:** **The segmentation algorithm parses each game to exploitation/exploration phases using the time differences between two consecutive gallery shape choices.** Shown are the segmentation results for several games. Each exploitation phase is marked by a different color. Exploration states are marked by a grey shaded area.

**Figure C:** **The ‘odd-shape-out’ test.** **People correctly recognized similarity of shapes within an exploitation phase.** The figure compares the distributions of bootstrapped ‘odd-out’ selection-ratio in the experiment and by chance. In the experiment participants choose the correct ‘odd-out’ shape ~45% of the time. Bootstrapping this choice-rate 10,000 times (sampling with returns) resulted in the right (‘Experiment’) histogram. A random choice out of four possible answers should resulted in a 25% ‘odd-out’ selection-ratio. The same bootstrapping procedure resulted in the left (‘Chance’) histogram, which is separated from the experimental results.


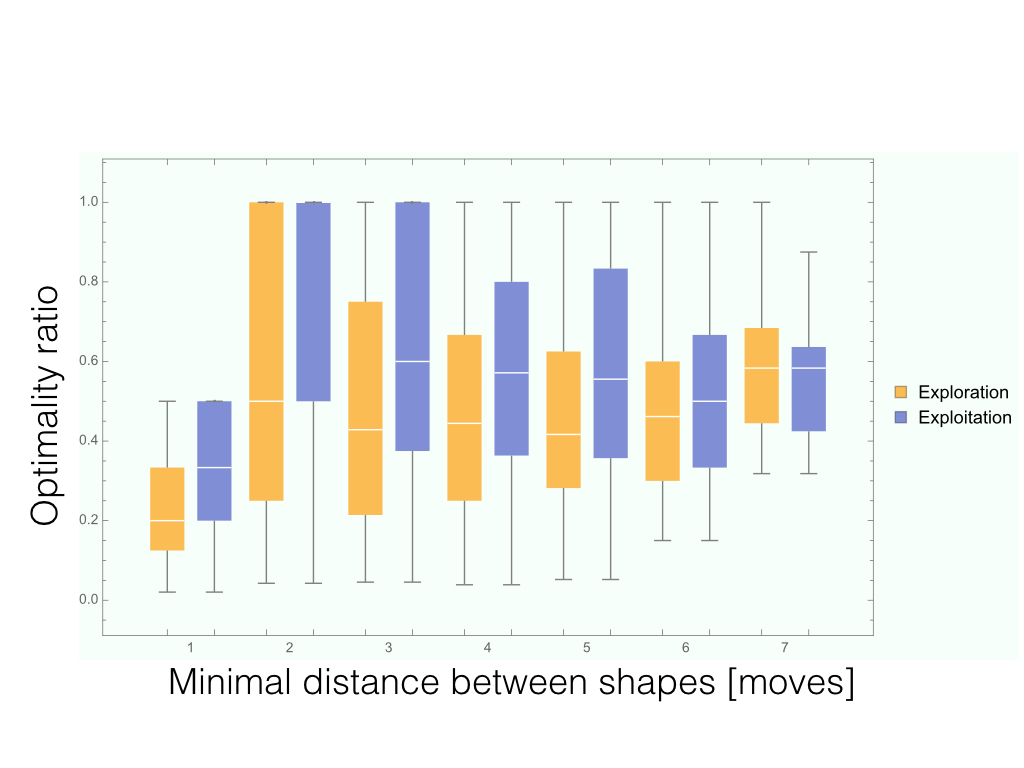


**Figure D:** **Ratio of minimal-to-actual path in the two phases per minimal path length.** Paths between two gallery shapes were binned according to the minimal number of possible moves needed to travel between the two shapes. In each bin, we show the distribution of this ratio (central point = median, boxes are 25%-75% quantiles) for exploration (gold) and exploitation (purple) phases. A ratio of 1.0 represents an optimal path – the actual path is equal to the minimal path, while smaller numbers represent meandering paths, were the actual path is longer than the minimal path. A lower minimal/actual ratio in exploitation phase is evident in bins 1-6.

**Figure E: Different players find shapes from the same categories A) Representative shapes from the four biggest categories**. Above each shape is its number of appearances in different patches. **B) People correctly identify groups of shapes from the same category.** Top: example of a test question. Bottom: correct choice rates, error bars by bootstrapping.


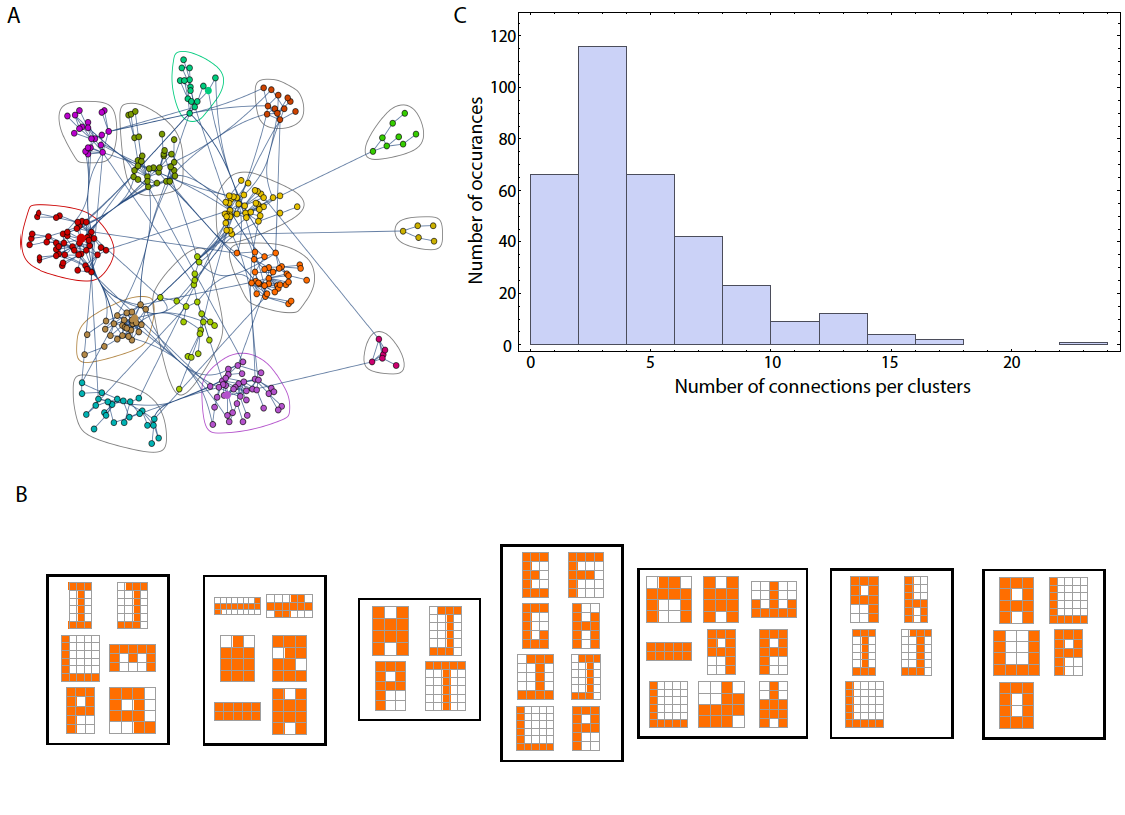


**Figure F: Patches combine to form categories with a joint theme.** A) The giant component of the network of patches has 14 categories (modules, communities). Each dot is a patch; patches in each category are color coded; categories are outlined. B) Example of patches with a connection (two shared shapes) between them within the ‘English letter’ category. C) Histogram of the number of connections of each patch. More than 61% of the patches are linked by at least four connections, indicating many shared shapes with other patches.


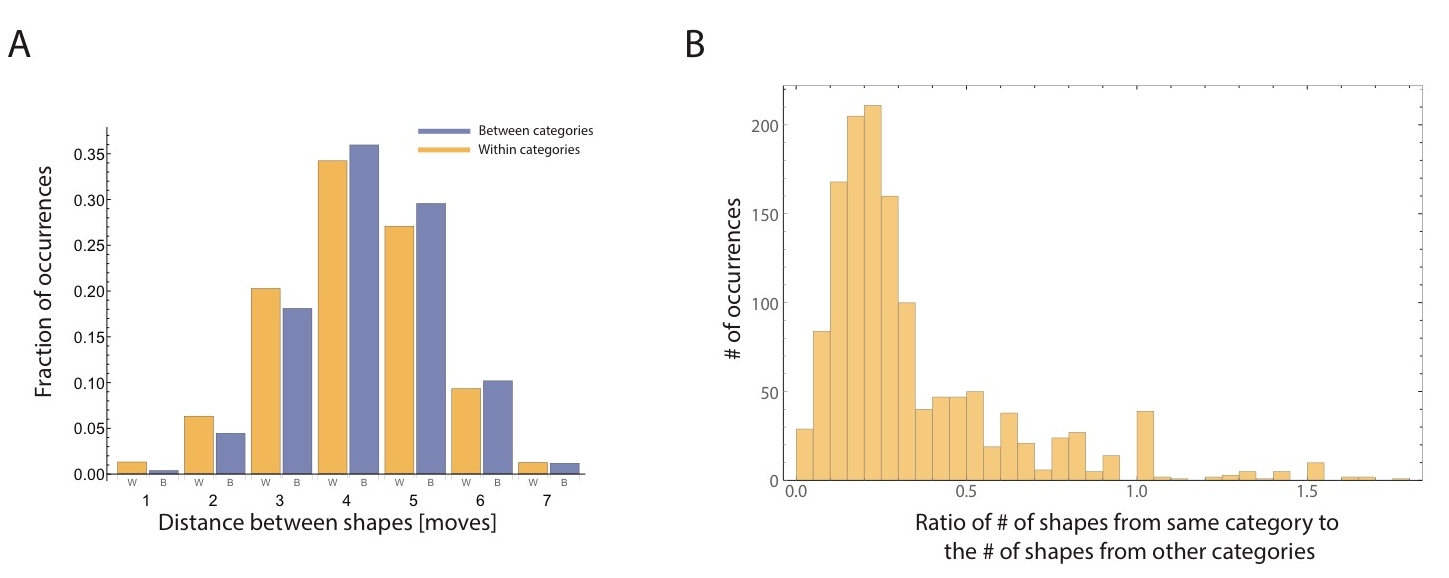


**Figure G: Meaning categories are interleaved.** **A)** **Equal distance distributions between shapes within a category and between categories.** Distance between two shapes within the same category (gold) and between different categories (purple) show high similarity with equivalent medians of 4 and 95% CI=[4,4]. **B)** **Within an exploitation phase, shapes from other categories are as equally distant as shapes from the same category.** We calculated the number of shapes from the same category of a shape chosen in the exploitation phase and compared it to the number of shapes from other categories available at the same distance (number of moves). The median ratio is about 0.25 which means that for every shape from the same category as the current shape there are 3 other possible shapes that are equally reachable.


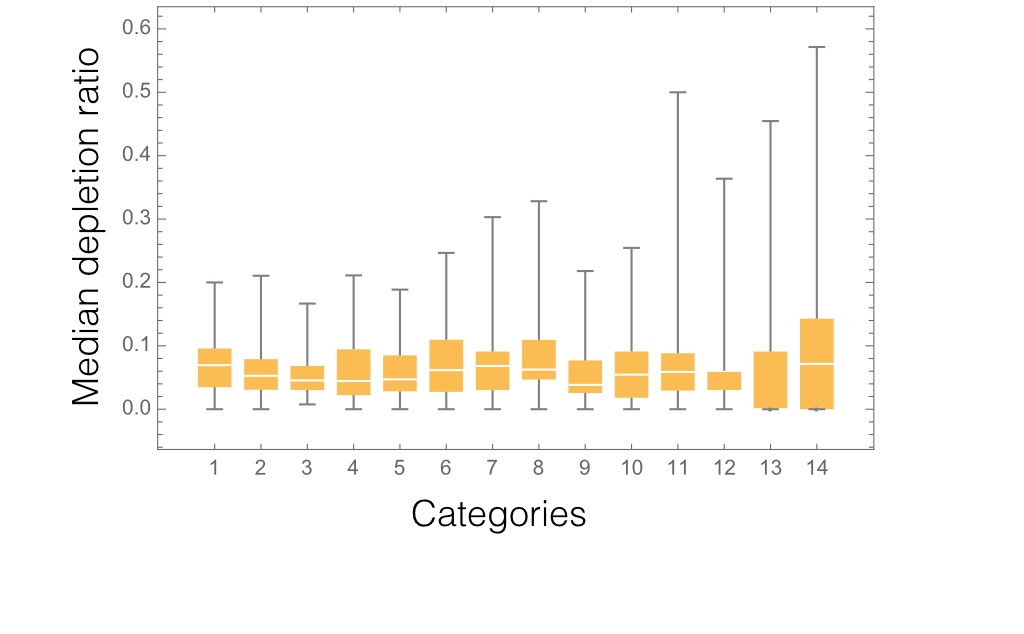


**Figure H: Categories are not depleted when players move to exploration phase.** We calculated the median across all players of the fraction of shapes covered from a specific category before leaving it and resuming exploration phase. The histogram of these medians per categories is presented. White lines are the medians, gold boxes delimit 25%-75% quantiles and thin lines indicate the min and max of each distribution. Note that high variability exists only in categories with low number of patches and shapes.


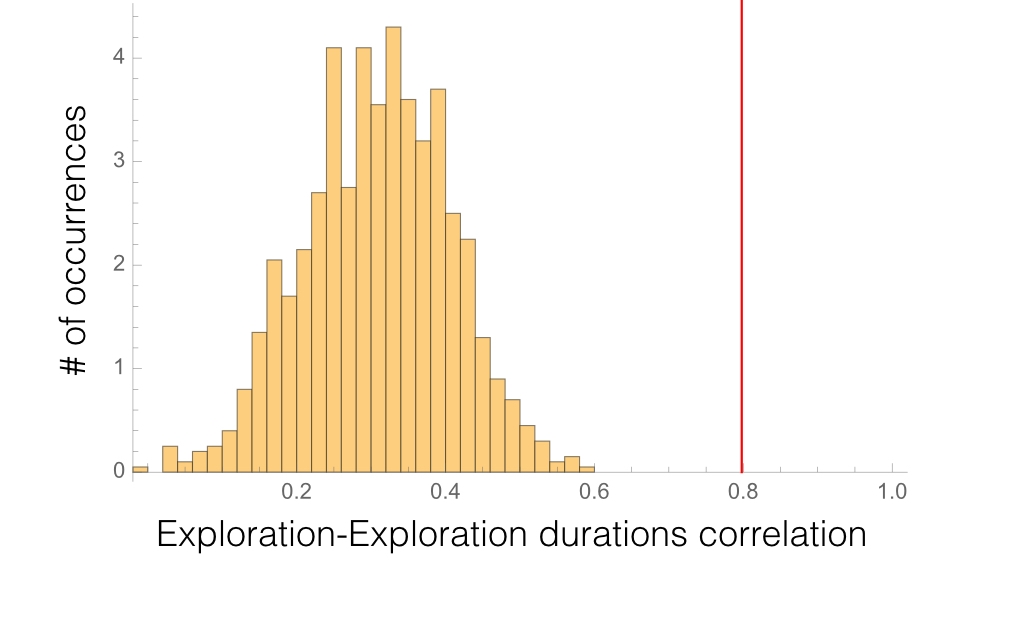


**Figure I: Exploration-Exploitation durations correlation does not stem from the segmentation algorithm or the timing distributions of each phase alone.** We build 100 artificial games, where timing between each shape was sampled from the distribution of timings between shapes. We then employed the segmentation algorithm to create exploration and exploitation phases and calculate the correlation between the mean durations of the two phases. We repeated this process 1000 times. We find that the correlation obtained from the real data does not overlap with the bootstrapped results, indicating p<0.001.

**
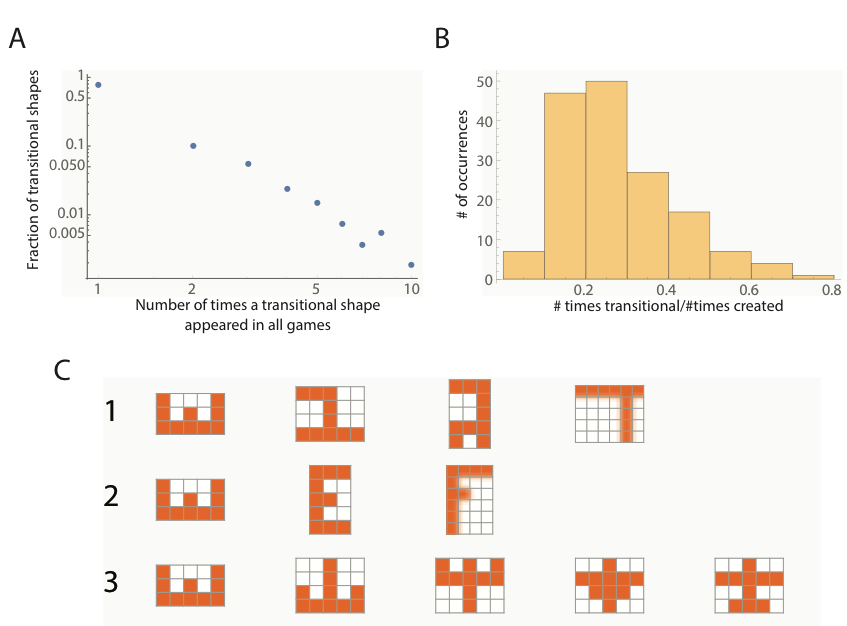
**

**Figure J: Transition shapes characteristics. A) Transition shapes are often unique to a player.** The frequency of games in which a shape was transitional (counting shapes that were transitional in at least one game). 77% of the transitional shapes are unique to a given player in a given game (intersection point with the y axis). **B)** **Transition shapes are overlooked by other players.** The distribution of the ratio of the number of times a shape was transitional compared to the number of times it was created (for shapes that were created at least 4 times and were transitional at least once). The median of the distribution is 0.23 indicating that there is a 1:4 chance a shape that was transitional for one player would be transitional to other players that find the shape. **C) Examples of different patches emerging from the same transition shape.** Different trajectories of gallery shapes were created by different players starting from the rotated ‘E’ shape, leading to different patches. 1) A sequence of Latin letters, going from a rotated ‘E’ to a regular ‘E’. 2) A sequence of Hebrew letters, starting with the Hebrew letter ‘Shin’ (ש/). 3) A sequence of shapes that may be described as ‘airplanes’.

# Tables

**Table A: Characteristics of shape categories**

|  | Number of patches | Number of connections within the category | Number of connections to other categories | Number of shapes | Number of unique shapes |
| --- | --- | --- | --- | --- | --- |
| **1** | 48 | 105 | 29 | 115 | 55 |
| **2** | 40 | 82 | 23 | 114 | 58 |
| **3** | 40 | 70 | 15 | 132 | 63 |
| **4** | 34 | 74 | 16 | 90 | 47 |
| **5** | 32 | 46 | 24 | 106 | 51 |
| **6** | 27 | 76 | 27 | 73 | 30 |
| **7** | 25 | 37 | 10 | 66 | 31 |
| **8** | 21 | 22 | 15 | 64 | 25 |
| **9** | 20 | 33 | 14 | 78 | 46 |
| **10** | 16 | 25 | 10 | 55 | 27 |
| **11** | 12 | 20 | 9 | 34 | 15 |
| **12** | 8 | 8 | 1 | 33 | 20 |
| **13** | 6 | 11 | 2 | 11 | 5 |
| **14** | 5 | 4 | 1 | 14 | 3 |

**Table B: Classifications of groups of shapes belonging to the same category.**

|  | % Correct | Description |
| --- | --- | --- |
| C1 | 86 | Hebrew Letters |
| C2 | 79 | English Letters |
| C3 | 73 | "Airplanes'" |
| C4 | 87 | "Spaceships" |
| C5 | 80 | "Compact shapes" |
| C6 | 67 | "Symmetric shapes" |
| C7 | 84 | Digits |
| C8 | 82 | "Space Invaders" |
| C9 | 80 | "Birds & Locks" |

# Appendix – complete written instructions for the experiment.

The next task is a game called “the search for the shifting shape”.

Your goal is to explore the world of shifting shapes and discover those you consider as interesting and beautiful.

The shapes are composed of cubes that touch each other. In each stage you can move one cube in order to change the current shape to the next one. A cube can be placed only in adjacent to another cube (not in diagonal), and it is not possible to remove a cube from the center of the shape. See an example of a possible and non-possible move in the next drawing:


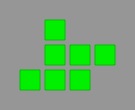


Current shape


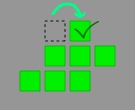


Possible move


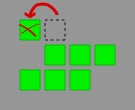


Non-possible move

Despite the simple rules of the game there are many beautiful and interesting shapes to discover. Your task is to move in each stage one cube in order to reach a new shape. You can save the shapes that you like in particular in the ‘Shape Gallery’, by clicking the red button at the top-right corner of the screen. You have to choose at least five shapes to the gallery.

In the last stage of the game you will be able to see all the shapes in the ‘Shape Gallery’ and will be asked to choose the ones you like most. The length of the game is 15 minutes.

Thanks for your cooperation! (press the link at the bottom of the page to continue)


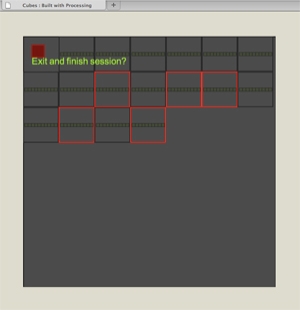


The last screen in which you choose among the gallery shapes


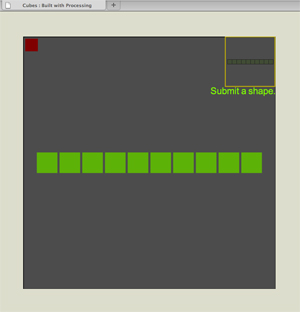


The screen during the game
